# Engineering Precision Medicine

**DOI:** 10.1002/advs.201801039

**Published:** 2018-10-25

**Authors:** Wujin Sun, Junmin Lee, Shiming Zhang, Cole Benyshek, Mehmet R. Dokmeci, Ali Khademhosseini

**Affiliations:** ^1^ Department of Bioengineering University of California–Los Angeles Los Angeles CA 90095 USA; ^2^ Center for Minimally Invasive Therapeutics (C‐MIT) California NanoSystems Institute University of California–Los Angeles Los Angeles CA 90095 USA; ^3^ Department of Radiology University of California–Los Angeles Los Angeles CA 90095 USA; ^4^ Jonsson Comprehensive Cancer Center University of California–Los Angeles 10833 Le Conte Ave Los Angeles CA 90024 USA; ^5^ Department of Chemical and Biomolecular Engineering University of California–Los Angeles Los Angeles CA 90095 USA; ^6^ Center of Nanotechnology Department of Physics King Abdulaziz University Jeddah 21569 Saudi Arabia; ^7^ Department of Bioindustrial Technologies College of Animal Bioscience and Technology Konkuk University Seoul 05029 Republic of Korea

**Keywords:** biomaterials, cell engineering, organs‐on‐chips, personalized devices, personalized implants

## Abstract

Advances in genomic sequencing and bioinformatics have led to the prospect of precision medicine where therapeutics can be advised by the genetic background of individuals. For example, mapping cancer genomics has revealed numerous genes that affect the therapeutic outcome of a drug. Through materials and cell engineering, many opportunities exist for engineers to contribute to precision medicine, such as engineering biosensors for diagnosis and health status monitoring, developing smart formulations for the controlled release of drugs, programming immune cells for targeted cancer therapy, differentiating pluripotent stem cells into desired lineages, fabricating bioscaffolds that support cell growth, or constructing “organs‐on‐chips” that can screen the effects of drugs. Collective engineering efforts will help transform precision medicine into a more personalized and effective healthcare approach. As continuous progress is made in engineering techniques, more tools will be available to fully realize precision medicine's potential.

## 1 Introduction

Each person responds differently to certain drugs. When prescribed with ineffective drugs, the patients may not only pay for the financial cost, but also suffer from the physiological side effects that could be catastrophic in certain circumstances.^1^ This calls for the tailoring of therapies to fit each individual. Customizing healthcare services for an individual or a group of people have the potential to reduce the economic burdens and enhance quality of life.

The “Precision Medicine Initiative” spearheaded by the Obama Administration proposed to apply the technological advances in genomics to improve healthcare outcomes.^2^ In 1990, the “Human Genome Project,” aimed at mapping an entire human genomic sequence, took about 13 years to complete, even with the collective efforts of scientists and engineers all around the world, and cost up to 1 billion US dollars.^3^ Since then, the “1000 Genomes Project” has mapped genomic sequences in 2504 individuals from 26 different global areas and identified over 88 million common genetic variations, including single nucleotide polymorphisms, insertions/deletions, and structural variants.^4^ With the tremendous progress made in sequencing techniques, genomic sequencing has become readily available to the general public, costing about $1000.^5^ There is no doubt that progress in next‐generation sequencing and genome‐wide association analysis will lead to the prospect of “precision medicine” where health care plans will take individual genomic variations into consideration.^6^ So far, over a million of human genomes have been sequenced in research settings,^7^ and this access to personal genomic data as well as our understanding of the genetic mechanisms of diseases has fueled the current concept of “precision medicine.”^8^

Genetic profiling, once an anecdotal technique in molecular biology, has become an easily accessible tool for the general public. Commercial genomic profiling companies, such as 23andMe that use saliva as the genetic source,^9^ not only provide the public with bioinformatic services but also inform people about genomic‐guided healthcare knowledge. Genomics‐based precision medicine can be applied to people of all ages, ranging from newborn infants to the elderly.^10^ Oncology is currently the main target for precision medicine due to the prevalence and lethality of cancer as well as the damaging side effects of anticancer therapies.^11^ Individual genetic mutations could be taken into account for predicting risk factors for cancer, guiding diagnostic tests, and designing treatments. Genomic profiling also casts light on the probability of cancer metastasis^12^ and tumor relapse^13^ where the doctor can take a biopsy from the patient for analysis. Precision medicine is a powerful approach and can benefit all types of cancers, and has been used in many cancers including pancreatic cancer,^14^ breast cancer,^15^ glioblastoma,^16^ anaplastic thyroid carcinoma,^17^ bladder cancer,^18^ colorectal cancer,^19^ biliary tract cancer,^20^ and adrenocortical tumors.^21^ Additionally, genetics‐based precision testing has also been incorporated into treating other illnesses such as newborn screening,^22^ pediatric rheumatology,^23^ cardiovascular disease,^24^ diabetes,^25^ hypertension,^26^ allergies,^27^ anaphylaxis,^28^ kidney disease,^29^ Parkinson's disease,^30^ multiple sclerosis,^31^ inflammatory bowel disease,^32^ and psychological diseases like suicidality^33^ and schizophrenia.^34^

Genomic sequencing has the potential to provide us with the information about genetic variations between people, though our understanding of the association between genetic sequences and diseases is far from being complete.^35^ In the simplified case of monogenic diseases, where a mutation in one gene causes a disease, the accuracy of diagnosis based only on genetic sequence information is unsatisfactory.^36^ This is due to our poor understanding of gene regulatory mechanisms.^37^ In the case of cancer, the ability of the cancer cells to mutate continuously makes it even more difficult to predict genotypes.^38^ Even with an improved understanding of the correlation between genotypes and phenotypes, the origin of many diseases cannot be explained by the information we can extract from genetic sequences.

While genomic profiling has been hailed as the driving force for precision medicine, surprisingly, little attention has been paid to engineering approaches. Even in the absence of genetic information, engineering can contribute to precision medicine by harnessing other aspects of personal health‐related information. For examples, without genomic profiles of a patient at hand, induced pluripotent stem cells (iPSCs) derived from the individual contain all the person's genomic information and they can be derived into various lineages to show patient‐specific responses to therapies. Without sequencing an individual's leukocyte antigens, patient‐derived immune cells could be engineered to target diseased cells without harming “self” cells of a patient. For information that cannot be predicted by genomics, such as such as heart rate, blood pressure, levels of metabolites, and biomarkers, engineered biosensors could provide accurate readout and provide a timely advice for medication. Drug delivery devices and regenerative therapies based on the engineering of smart biomaterial can respond differently to an individual's physiological traits for realizing personalized therapies.

**Figure 1:**
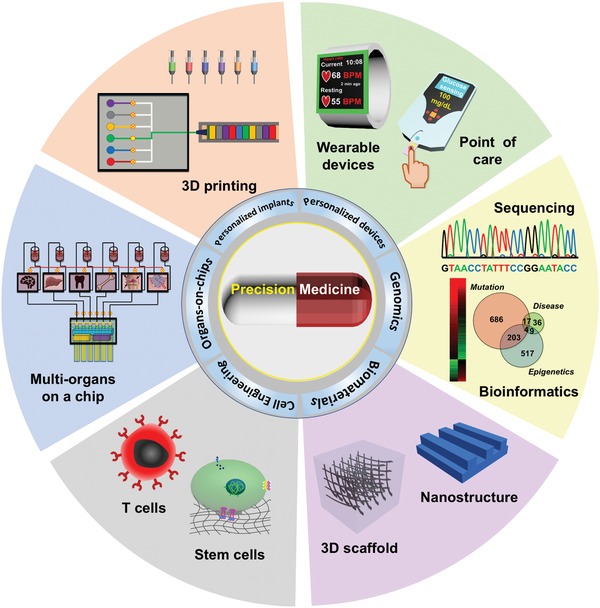
Engineering precision medicine. Biomaterials engineering, cell engineering, organs‐on‐chips, personalized implants, and personalized devices together with genomics‐based methods to enable precision medicine.

In this review, we will discuss engineering approaches that consider both genetic and nongenetic factors to provide higher treatment precision (**Figure** **1**). More specifically, we will describe personalized biomaterials that could provide scaffolds for artificial tissue growth and tools for surgical intervention, biomaterial‐based drug delivery systems that could provide on‐demand drug release by integrating sensing and drug release in a closed‐loop system, wearable medical devices that could monitor the physiological status of a patient in real time, engineered immune cells that could directly harness the internal defense system of a patient to fight diseases, stem cells that could be developed using patient‐sourced cells to provide patient‐specific cells or organs, and “organs‐on‐chips” with patient‐derived cells that could provide direct information about individual responses to prescribed therapies. Furthermore, we will discuss emerging technologies that could further contribute to precision medicine and the challenges that need to be addressed for this technology to reach its full potential.

## 2 Engineering Precision Medicine

### 2.1 Engineering Cells for Precision Medicine

#### 2.1.1 Immune Cell Engineering for Cancer Therapy

Human immune system maintains the health of the body by fendering out exogenous pathogens as well as clearing out endogenously failed cells. There have been century‐long efforts to harness immune systems for human health, and vaccination has been a standard healthcare method in the last few decades.^39^ Recent breakthroughs in cancer immunotherapy, like discovering immune checkpoints and engineering chimeric antigen receptor (CAR)‐T cells,^40^ have reinvigorated the field of oncology. Immune therapies harnessing patients' immune systems, including phenotypically activating or genetically engineering autologous immune cells, provide another approach for tailoring precision medicine.

Vaccines achieve precision medicine by using a fragment of disease‐relevant peptide to train immune cells, especially B cell and T cells, to recognize the target cells. The long‐recognized potency of vaccines in preventing infectious diseases has inspired physicians to acquire a cancer‐specific vaccine.^41^ The way of training the patients' own immune cells to recognize cancers using vaccines is straightforward, but there exist many more hurdles for cancer vaccines than the ones for targeting viruses due to the fact that cancer cells have many antigens similar to normal cells.^42^ Mutated proteins which are characteristics of specific cancers, namely neoantigens, are the rare markers that could distinguish one cancer from another.^43^ Key to the vaccination strategy is the identification of patient‐specific cancer neoantigens, and promising results have already been attained for several skin associated cancers.^44^ An emerging strategy to detour the tremendous efforts for neoantigen screening, however, is to instead use the whole cell lysate of the patient‐derived tumors for stimulating the immune system.^45^

**Figure 2:**
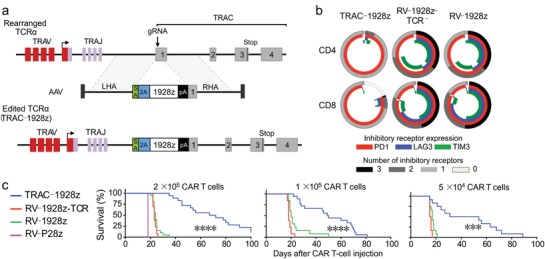
Engineering CAR‐T cells using CRISPR‐Cas9 for precision cancer immunotherapy. a) The CD19 CAR was precisely inserted into the TRAC locus by CRISPR‐Cas9‐mediated genome editing. The insertion also caused the knockout of T cell receptors, delaying T cell exhaustion. b) Quantification of T cell exhaustion markers expression on the engineered T cell. Insertion of CAR into the T cell receptors locus (TRAC‐1928z) significantly reduced the expression of T cell exhaustion markers, showing huge advantage over non‐targeted construction approaches. c) The 2‐in‐1 engineering strategy generated CAR‐T cells with more robust anticancer efficacy, increasing the survival of treated mice. Reproduced with permission.^48^ Copyright 2017, Springer Nature.

Training a patient's immune system to fight cancer is a multistep biological process where the therapeutic efficacy is difficult to predict. In comparison, synthetic biology approaches can be used to directly reprogram a patient's immune cells to recognize a signature antigen on cancer cell surfaces. This provides a faster and more controllable way of activating immune systems.^46^ T cells can directly kill cancer cells when the T cells and the costimulatory receptors recognize the antigens on target cell membranes.^47^ A simplified version of the T cell receptors—CAR—was engineered into patient‐derived T cells to recognize a predefined cancer antigen in that patient. In an improved construct, Eyquem et al. inserted the CAR‐encoding DNA fragment to the genomic locus of T cell receptor α constant (TRAC) with the assistance of CRISPR‐Cas9 (**Figure** **2**a), leading to much higher potency than viral vector‐constructed counterparts (Figure 2b). Robust anticancer efficacy of the CAR‐T cells has been demonstrated that significantly improved the survival of treat mice (Figure 2c).^48^ In 2017, the first two CAR‐T therapies, Yescarta and Kymriah, that target CD‐19 of B‐cell leukemias, were approved.^49^ Hundreds of more CAR‐T cells are under development for many cancers,^50^ among which both liquid hematological cancers and solid tumors, such as pancreatic cancer, ovarian cancer, glioblastoma, neuroblastoma, are included.^51^ However, the immunosuppressive tumor microenvironment, strong stroma barrier, and genotype mutations of the cancer cells in solid tumors made them more difficult to be killed.

The potency of CAR‐T therapy toward blood cancer cells is accompanied by safety concerns, such as off‐target binding to healthy cells or the occurrence of cytokine storms, that could be lethal in some cases.^52^ Thus, more engineering approaches are needed to precisely control the activity of injected CAR‐T cells, like turning on CAR signaling with external signals^53^ or causing the suicide of the CAR‐T cells in the case of cytokine storm.^54^ The success of the engineered T cells also inspired arming nonimmune cells, such as human embryonic kidney cells, with cancer sensors and killing effectors for targeted tumor therapy.^55^ This nonimmune cell engineering strategy could reduce the concern for unexpected immune cell activation.

By obviating the necessity of genetically engineering T cells, immune checkpoint inhibitors can directly activate a patient's T cells for fighting cancer. Immune checkpoint molecules are expressed on immune cells as tolerance regulators to minimize the potential damage to normal tissues.^56^ This pathway becomes hijacked by cancer cells or other pathogens to evade T cell‐mediated immune surveillance or even “exhaust” T cells into nonfunctional states.^57^ Programmed cell death protein 1 (PD1) and cytotoxic T lymphocyte antigen 4 are two well‐characterized immune checkpoints where antibodies that target these pathways have demonstrated robust therapeutic efficacy in treating multiple types of cancer. Several PD1 or Programmed Death‐Ligand 1 inhibitors, such as avelumab,^58^ nivolumab,^59^ atezolizumab,^60^ pembrolizumab,^61^ and durvalumab,^62^ have received accelerated approval from FDA for treating melanoma, non‐small‐cell lung carcinoma, head and neck squamous cell carcinoma, bladder cancer, and metastatic urothelial carcinoma.^63^ This approach has the advantage of harnessing endogenous T cells in their natural state, avoiding the time consuming and expensive process of engineering T cells. In terms of precision medicine, typing the dominant immune checkpoint pathways in patients' tumors could help guide oncologists to decide the optimal inhibitor for each patient. The discovery of new immune checkpoint pathways will thereby further expand this therapy, helping more patients with precision.

#### 2.1.2 Stem Cell Engineering for Precision Medicine

**Figure 3:**
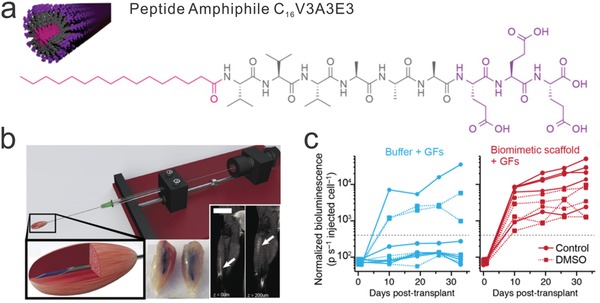
Bioscaffold‐mediated stem cell delivery for muscle regeneration. a) Nanofibers self‐assembled from amphiphilic peptides were designed to encapsulate muscle stem cells and growth factors to facilitate muscle regeneration. b) Injection of the stem cell‐loaded scaffold into the target location using a delivery device. c) Muscle stem cells delivered by the injectable scaffold showed more robust proliferation. Reproduced with permission.^66^ Copyright 2017, National Academy of Sciences.

Human pluripotent stem cells derived from embryos can self‐replicate in vitro indefinitely and differentiate into almost any desirable cell types.^64^ Due to the ethical and availability issues associated with using embryonic stem cells, iPSCs are used as an alternative. iPSCs provide a more accessible source of stem cells for regenerative medicine^65^ and could generate cell replacement/regeneration therapies. Stupp and co‐workers demonstrated a scaffold based on self‐assembled peptides to mimic the unidirectional structure of muscle fiber (**Figure** **3**a).^66^ With growth factors and muscle stem cells encapsulated in the fibers, the stem cell therapy was delivered by a retracting injection system (Figure 3b). The peptide base scaffold significantly improved the engraftment efficacy of muscle stems cells (Figure 3c). Furthermore, iPSCs could also be used to build a “disease‐in‐a‐dish” model for disease modeling or drug screening.^67^ Since the iPSCs can be obtained from patient‐derived cells, such as skin or blood, they contain the potential of enabling personalized therapies or disease models with exactly the same genomic background as the patient. With the advances in recent genome editing technologies, cells with genetically desirable phenotypes, such as the ones expressing desired biomarkers, could be easily generated.^68^ Personalized cells with rationally engineered genetic traits provide a versatile platform for generating precision medicine therapies.

Stem cell‐based precision medicine could be attained through the following scenarios: 1) healthy cells from patients with failed organs could be reprogrammed into iPSCs, and the iPSCs redifferentiate into desired cell types, such as cardiomyocytes^69^ or neurons,^70^ for refilling the degenerated organs. For example, iPSC‐derived eye‐associated cells (retinal pigment epithelial) were among the first that was translated to the clinical trials.^71^ 2) iPSC with patient‐derived genetic defects, like monogenic disorders, can be genetically corrected by gene editing technologies. The engineered stem cells could then be used as the source for growing healthy cells for cell replacement or repair therapy.^72^ 3) iPSCs with patient‐specific genotypes can be directly differentiated into the disease‐associated cell types for disease modeling. This application can be useful in cases where the cause of the disease is unclear, and direct screening for an effective therapy is not possible.

However, the iPSC‐based engineering approach could be time‐consuming. Recent developments in cell reprogramming enabled the iPSC stage to be skipped and had one type of somatic cells directly converted into another type of somatic cells. This process is known as transdifferentiation.^73^ This transdifferentiation approach has been applied to convert fibroblasts into other cell types including cardiomycytes,^74^ neurons,^75^ or hepatocytes.^76^ Nonetheless, skipping the iPSC stage also skipped its proliferation capabilities, making scaling‐up difficult. Stem cells can be delivered in the form of scaffold‐free cells or with the assistance polymeric scaffolds. Soft biocompatible materials are generally used as scaffolds, providing a niche to mimic the mechanical properties of the natural extracellular matrix (ECM). These scaffolds can be integrated with signaling cues to regulate the proliferation, differentiation, and migration of the cells in a way similar to tissue engineering approaches.^77^

### 2.2 Engineering Precision Biomaterials for Tissue Engineering and Drug Delivery

Advances in biomaterials contribute to precision medicine by providing a toolbox of biocompatible materials that can interact with cells and tissues in a predictable manner.^78^ Studies of material–cell interactions provide us with knowledge about the effects of the environment on cells as well as give us the tools to engineer tissue scaffolds, medical devices, and therapeutic delivery carriers.^79^ Precision biomaterials are engineered with specific mechanical and biochemical properties to enable the design of personalized implants or organ replacements. For example, shear‐thinning polymers could be easily injected and conform to the shape of a patient's cavity to form a patient size specific implant;^80^ enzyme or pH degradable materials can be used in manufacturing bioscaffold that will be resorbed by the patient in a rate specific to the patient's physiological environment; decellularized ECM from a patient‐derived tissue provides a highly biocompatible as well as size‐fit scaffold. To build complex smart devices for biomedical engineering, precision biomaterials need to be engineered with the ability to execute numerous unit operations, such as “separator” where the material can specifically attach to molecules or cells for separation, “sensors” where the material can detect specific analytes or electrochemical signals, “responders” where the material can change its morphology or get degraded to release the payload, “controllers” where the material can affect local microenvironment for changing the behavior of the cells, “processors” where the material allows high throughput analysis.^81^

Progress in polymer chemistry has improved our control over polymerization, and the versatility in polymer chemical composition tuning has generated a large library of biocompatible materials, such as polyethylene glycol, polycaprolactone, poly (N‐isopropylacrylamide), poly(glycolic acid), poly(lactic acid), and poly(lactic acid‐co‐glycolic acid). Other than synthetic materials, the modification of natural compounds, ranging from proteins to polysaccharides, has resulted in many highly biocompatible materials.^82^ For example, gelatin methacryloyl is a widely used material that mimics many properties of the ECM, including protease‐assisted degradability, tunable mechanical strength, and easy functionalization, to support cell attachment, proliferation, and migration.^83^ Other well‐characterized natural polymers include elastin, collagen, chitin, chitosan, alginate, and hyaluronic acid.^84^ Additionally, these naturally derived materials also have the advantage of showing minimal inflammatory responses even after long‐term implantation.

**Figure 4:**
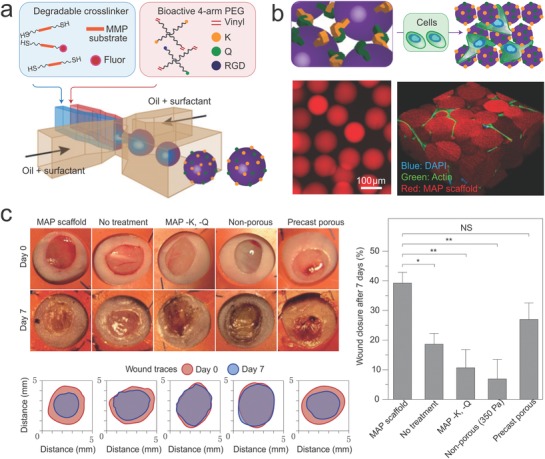
A porous and degradable scaffold for personalized wound treatment. a) Polymeric microparticles were prepared by microfluidics, incorporating a metalloprotease‐degradable peptide as cross‐linkers. The water‐in‐oil approach segmented the monomers and crosslinkers into monodispersed droplets, which were then crosslinked inside the droplets. b) The microparticles were further purified and annealed with FXIIIa to form porous scaffolds that can support cell growth. c) The induction of in vivo wound healing by the engineered scaffold for 7 days. Shown are the representative wound healing images from Balb/c mice. The microparticle‐based porous scaffold induced much faster wound healing than other materials. Reproduced with permission.^89^ Copyright 2015, Springer Nature.

Development in bioconjugate chemistry has facilitated our ability to synergize the desired properties of different materials, such as the modification of nonadherent polymeric scaffolds with the RGD peptide for cell attachment^85^ and inclusion of various growth factors or morphogen for directing cell growth.^86^ It has been shown that tuning the physical or biochemical properties of biomaterials, such as matrix stiffness, topology, or the number and type of adhesive ligands, could direct lineage specification of stem cells.^87^ By controlling these parameters, engineered biomaterials can simulate the ECM of different organs and could help the growth of respective artificial organs. For example, by using a collagen‐based scaffold, an autologous bladder has already been created using patient‐derived biopsies for transplantation.^88^ Furthermore, by utilizing an injectable microporous scaffold that has a degradation rate determined by the wound‐environment‐associated metalloprotease of individual patients, patient endogenous cells have been recruited to wound sites for tissue regeneration (**Figure** **4**).^89^ The porous scaffold was assembled from microparticles prepared by microfluidics, where metalloprotease‐degradable cross‐linkers were incorporated (Figure 4a). Robust cell growth and migration could be observed within the porous structure (Figure 4b) and rapid tissue regeneration for wound‐bed closure was observed in a mouse model (Figure 4c).

In addition to supporting the growth of artificial tissues, biomaterials alone can be tailored for direct in vivo tissue interventions. For instance, an angiographic catheter made of textured nylon has been used to vascularize a subcutaneous space for human islet transplantation.^90^ Although the biomaterials were removed during the transplantation, preconditioning of the transplantation site with the biomaterials improved the viability of the islet cells. In another example, a shear‐thinning material tailored from gelatin and inorganic nanoparticles was demonstrated to intervene in blood flow.^91^ Delivered by a catheter, the hybrid biomaterial was used to achieve a minimally invasive method of targeted vascular embolization.

Another aspect through which biomaterials could help supplement precision medicine is through the engineering of smart drug delivery systems. Drug delivery devices could improve the “precision” of precision medicine through pharmacokinetics.^92^ Ideally, customized drug delivery systems could deliver the optimal dose of the drug to the intended organs at the specific time.^93^ Engineering smart biomaterials for drug delivery systems that can sense the needs of an individual's physiological state and adjust its drug release profile accordingly is an attractive strategy for formulating precision pharmacokinetics.^94^

Nanomaterial‐based drug delivery systems could penetrate multiple physiological barriers and provide a simple strategy for the targeted delivery of therapeutics to diseased sites.^95^ Both synthetic materials, such as polymers, lipids, inorganic salts, and metals, or natural materials, such as proteins, nucleic acids, or cell membranes,^96^ can be prepared as nanoscaffolds for this purpose, and with more advanced sensors and actuators, nanoscopic smart drug delivery systems could also be further engineered.^97^ In addition, advances in surface chemistry have brought about nanoscale drug delivery systems that can evade immune surveillance. Armed with targeting ligands, such as antibodies, peptides, aptamers, and small molecules, nanoscopic drug carriers could get continually delivered to desired tissues after extended in vivo circulation.^98^ With the availability of a pool of effective and specific antibodies for targeting various plasma‐membrane associated antigens, profiling targetable antigens on diseased sites, like tumors, allows nanoparticle based drug delivery systems (nanomedicine) to be customized for each patient.^99^ To ensure that the loaded drug will only be released at the targeted site, the nanocarriers will need to hold the drug until they have reached the designed destination. To achieve this, physiological characteristics associated with the disease, such as pH change, overexpressed hydrolytic enzymes, increased reducing or oxidative environments, or the variations in oxygen levels, could be used as cues for the release of the drugs.^100^ Specific drug release could also be controlled using external physical signals, such as ultrasound, magnetic fields, electric fields, or radio‐waves.^101^ These cues could be used either individually or synergistically to improve the accuracy and timeliness that will efficiently deliver drugs to targeted tumors. With the progress already made in conjunction with a comprehensive list of stimuli‐responsive materials, a variety of smart drug delivery systems can be constructed.^102^ The emerging field of engineering nanoscale theranostics that integrate diagnostic and therapeutic modules into one nanoformulation provides real‐time visualization of drug delivery processes.^103^ This combined approach allows for the visualized optimization of nanomedicine therapeutic efficacy that can then be applied into the different physiology of each patient. When nanocarriers are combined with gene therapies, such as plasmid DNA, mRNA, iRNA, or miRNA, the nanomaterial formulation could be tailored to meet the individual needs of the patient based on their specific genetics.^104^

**Figure 5:**
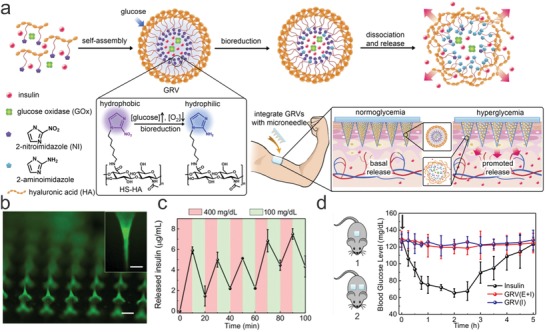
A closed‐loop drug delivery system that enables precise dosing of insulin according to the blood sugar level of the patient. a) Schematics of the drug delivery device; polymeric micelles that could release insulin in response to blood sugar levels were formulated into microneedles for painless delivery. b) Fluorescent microscope image of the microneedle patch. c) Glucose‐responsive insulin release from the microneedle patch in vitro. d) The insulin delivery patch kept blood glucose levels within a normal range for an extended range of time. Reproduced with permission.^108^ Copyright 2015, National Academy of Sciences.

Besides systemically administered formulations that utilize targeting ligands for achieving “precision,” local delivery has the advantage of directly acting on the desired tissue.^105^ For chronic diseases that have a constant requirement of a certain level of drug in the blood, administering repeated drug doses to the patient is important for maintaining the drug level within the therapeutic window to enhance the effectiveness of the applied medicine. Drug release depots may therefore be required for continuous local drug delivery, which would alleviate this issue by maintaining a therapeutically effective dosage over an extended time frame.^106^ In terms of “precision,” closed‐loop drug delivery systems that monitor the level of a physiological signal, such as the concentration of a molecule, and control the release of a drug accordingly fit this model.^107^ Gu and co‐workers demonstrated a “Closed‐loop” device that uses integrated sensors for sensing blood glucose levels and actuated insulin drug release from stored depots that are notable “precise dosing” devices (**Figure** **5**a).^108^ The formulation was delivered in the format of a microneedles patch (Figure 5b) and a glucose level‐dependent release of insulin release could be observed both in vitro and in vivo (Figure 5c,d). Besides blood glucose monitoring, this “closed‐loop” strategy is highly desirable in treating other diseases that need real‐time regulation of the drug dosage.

### 2.3 Smart Diagnostic Devices

#### 2.3.1 Point of Care Devices

Rapid analysis of biochemical markers in an individual enables precision medicine by providing rapid readout of physiological markers. Advances in smart sensors enable “point‐of‐care” (POC) devices that allow analyzing our health status at any time and at any place.^109^ It is defined as medical diagnosis performed at the site of care, i.e., at the time and place of patient care. Fingerstick blood test is a simple example of POC devices,^110^ they are designed either to give a digital result for pregnancy test or a numerical measurement for glucose concentration of the blood.^111^ The global POC device market is expected to grow from US$ 23.16 in 2016 to US$ 36.96 billion in 2021 at the compound annual growth rate of 9.8% from 2016 to 2021.^112^

Large diagnostic facilities in hospitals, including imaging techniques such as ultrasound,^113^ magnetic resonance imaging,^114^ and computed tomography (CT),^115^ provide valuable information about the status of organs in the patients. However, the equipment are large, and have a high time‐cost burden and require trained personnel. POC devices show significant advantages in spatial flexibility over the heavy medical equipment. Besides, POC testing has a much faster turnaround time. The spatial flexibility and timeliness of POC testing allow immediate diagnosis of diseases by rapid detection of patient‐derived samples, such as saliva, urine, or blood, and enable much quicker medical decisions.^112^ In such a way, diseases can be diagnosed and treated at very early stage. It reduces the possibility of further deterioration of the pathological condition, which in certain cases may lead to fatal risks such as heart failure, stroke, and cancer. POC devices are in huge demand from patients with chronic diseases and people whose physical condition requires inspection on a regular basis, such as for the elderly and postoperative patients. Furthermore, POC devices are labeled as more user friendly. This is of great importance for disease diagnostics in developing and underdeveloped nations where healthcare services are inadequate.

Current commercial POC devices are mostly designed to analyze blood or urine.^116^ The recent advances in flexible electronics, bioelectronics, and biosensors are extending the application of POC devices toward testing of biomarkers in sweat, tear, saliva, and interstitial fluid using electrochemical and microfluidic sensors; mechanical movements of the human body using piezoelectric devices; physiological signals such as electrocardiography, electromyography, and electroencephalography using flexible electronics. Moreover, the emerging technologies in biocompatible sensors are rapidly enabling POC testing of diseases directly inside our body by using ingestible POC devices that are composed of fully biocompatible materials. With advances in these technologies, we can vision more revolutionary POC devices to be developed.

#### 2.3.2 Wearable Devices

In order to know the biochemical characteristics of each individual for precision medicine treatments, real‐time sensing is necessary. Readily available and applicable sensing devices not only improve the capability of physicians to precisely diagnose a disease, but also increase the participation of patients where the patient could easily monitor their own health.^117^ In particular, the field of emerging wearable sensors allows people to monitor their health and know their specific healthcare needs in real time.^118^

**Figure 6:**
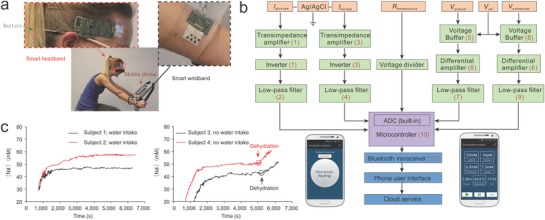
A wearable device integrated with multiple sensors to enable precise monitoring of the physiological status of an individual. a) The sensor can be worn on the wrist or forehead of an individual to monitor physical activities. b) The wearable band is designed to monitor glucose, lactose, sodium, and potassium levels in sweat as well as body temperature in real time. c) A proof‐of‐principle study to monitor subject's dehydration status from physical activities. Reproduced with permission.^120^ Copyright 2016, Springer Nature.

Portable and disposable sensors that can track physiological signals without the assistance of medical experts are thus highly desirable. Rapid monitoring of a patient's condition with simple devices provides valuable data that could be used for accurate evaluation of the patient's health status.^119^ More recently, wearable sensors and flexible electronics have shown great potential in making real‐time health monitoring devices. Similar to commercially available smartwatches that contain heart rate sensors as a standard configuration, wearable electronic devices that can monitor sweat biomarkers are emerging as key players in this realm. Gao et al. developed a sensor array that could be worn as wristband or headband to monitor metabolites in the sweat (**Figure** **6**a).^120^ The integration of sensors and wireless communication component enabled real‐time monitoring of perspiration‐related health information on mobile phones (Figure 6b). In a proof‐of‐concept study, the wearable sensors provide real‐time monitoring of a person's hydration status (Figure 6c). There are many other devices that integrate several sensors and can monitor multiple physiological signals in real time to provide a better perspective about the status of human body.^121^

With the integration of electrochemical sensors, wearable devices could detect health‐related biomarkers. These devices provide a noninvasive method that extracts health‐related information from bodily fluids. For example, monitoring biomarkers in interstitial fluid (ISF) gains comparable information with respect to blood monitoring because key biomarkers reflecting body condition such as glucose, ion concentrations (such as Na^+^, K^+^), proteins in the ISF are very similar to blood. Hence, in recent years, developing wearable sensors monitoring biomarkers in ISF has attracted considerable attention. It has the potential to replace conventional sampling from blood in a minimally invasive manner by using microneedles or iontophoresis electrodes.^122^ Saliva also contains a variety of biomarkers, including malondialdehyde, vitamin C, and proteomes, which are related to oxidative stress and thus have been used as biospecimen for the reflection of diseases such as autism, Alzheimer's disease, Parkinson's disease, atherosclerosis, heart failure, and cancer.^123^ Continuous monitoring of ionized calcium and pH of sweat using a wearable sensor provides essential information on human metabolism and minerals homeostasis.^124^ Proteins, salts, enzymes, and other chemical composition in the tear reflect the eye‐related diseases such as dye eye and systemic disorders of the human body. Tumor‐related biomarkers are also recently found in tear which can be used to predict breast cancer. Recording temperature, PH, and oxygen concentration in the wounded skin allows us to gain significant information on the wound healing process and helps the treatment of chronic wound by developing smart bandages that integrate with drug delivery systems.^125^

In addition, physiological signals can be detected in a noninvasive manner by attaching “skin‐sensors” onto the human body.^126^ For example, blood pressure is measurable by developing wearable sensors containing piezoelectric materials which are able to produce an electric signal when they are placed under mechanical stress. The pulses of the blood can hence induce a voltage signal in the piezoelectric materials and subsequently be collected by the sensor.^127^ Similar piezoelectric sensors have been used to detect heart rate, breath content, and other body motions.^128^ Recording electrocardiogram (ECG), electromyogram, electroencephalogram (EEG) signals can be realized by developing sensors that incorporate ionic gel electrodes or ion‐to‐electron transducers such as conducting polymers.^129^ These sensors are able to detect ionic electrophysiological signals of the human body and convert these ionic signals to processable electronic signals.^130^ Their low skin‐contact impedance and ability to amplify the weak ionic current of the physiological signals allow recording of the physiological signals such as ECG and EEG with high signal to noise ratios.^129^, ^131^

Devices that monitor glucose levels from sweat and use this information to trigger insulin release are available for diabetic patients.^132^ In this instance, however, the poor correlation between blood and sweat glucose levels could potentially limit the translation of this technology to skin sensors. Changes in sweat blood glucose levels have been shown to have 10–20 min delays.^133^ Similar delays may also be observed in other types of secreted body fluids, which can be potentially dangerous for diabetic treatments. In addition, the secretion mechanisms of many other biomolecules, such as proteins, peptides, or hormones, into body fluids are still unclear.^134^ Nevertheless, a promising solution to improve sensing accuracy is to combine these sensors with minimally invasive devices like microneedles, so that the resulting devices can directly monitor biomarkers contained in blood. Microneedles, which create small pores on the skin that cause little to no pain, provide a platform to incorporate interstitial fluid and blood biomarker‐targeted sensors.^135^ Furthermore, sensors integrated onto minimally invasive surgical tools for gathering internal tissue related signals could enhance the precision of surgical operations. For example, a sensor on a biopsy needle measuring young's modulus has been employed to help surgeons to monitor the mechanical properties of the tissues around the needle in real time.^136^ This way, surgery precision was improved by distinguishing between the mechanical properties of normal tissues from diseased ones, such as tumors.

Personal health data can be collected by these aforementioned sensors continuously. Intelligent devices like smart phones can be employed to learn the biomolecular patterns and lifestyles of the patients through wireless communication. Personalized healthcare advice or intervention could then be generated based on this information. With advances in sensing techniques, we expect that the sensor platform can not only monitor the physical and biochemical signals from the human body but can also analyze the data collected through the sensor so that it is possible to alert individuals about their psychological ailments, such as anxiety, stress, or depression in real‐time.^137^ In the future, a smartwatch may not only remind one to take the medicine, but also tell the person to calm down when one feels nervous (while giving a talk to a large crowd of people, for example) and directly alert medical staff when fatal signals, such as heart attack, are received.

### 2.4 3D‐Printing Facilitated Precision Tissue Engineering

As 3D printing technology advances, engineering medical implants and artificial organs with patient‐specific spatial architecture has emerged as an approach for precision medicine.^138^ Like inkjet printers, 3D printers have simplified the process of designing and manufacturing products using a range of different materials as the ink, such as polymers, ceramics, or metals.^139^ The 3D printing technology has been widely adopted into producing low volume end‐using parts. Numerous additive manufacturing methods, such as material jetting, binder jetting, material extrusion, vat photopolymerization, sheet lamination, powder bed fusion, and direct energy depostion, have been adopted into the production of medical implants.^140^ With patient‐derived anatomy information and clinically relevant biomaterials, implants with patient‐specific shape and size could be precisely manufactured by 3D printing.^141^ Customized implants that perfectly fit the defect sites of the patients can significantly shorten the time for surgical operation, reducing the need for adding fillers or removing healthy tissues. Calcium phosphate, bioactive glasses, and metals are commonly used “ink” in 3D printing of orthopedic implants.^142^ The 3D printed hips with customized size and sufficient mechanical strength after printing are still strong after a decade of implantation. The 3D printing technique is also handy in tailoring replacements for craniofacial defects, which often cause physiological and psychological pains to the patient. Reconstituting the complex 3D structure of the damaged craniofacial regions needs to be done with high structural precision due to the consideration of aesthetic outcomes.^143^ Besides hard material based implants, 3D printing is also powerful in manufacturing soft tissue implants, such as for lung and heart therapy. With computer tomographic image of a patient's airway, an artificial trachea splint has been laser‐printed with bioresorbable polymer.^144^ The customized implant has been used in treating tracheobronchomalacia in newborns, where continuous growth of the child requires the implant to be fit as well as elastic. Using a highly haemocompatible elastomeric material (silicone/polyurethane) as the ink, a soft occluder with CT‐imaged acquired patient‐specific size has been printed for the left atrial appendage.^145^

**Figure 7:**
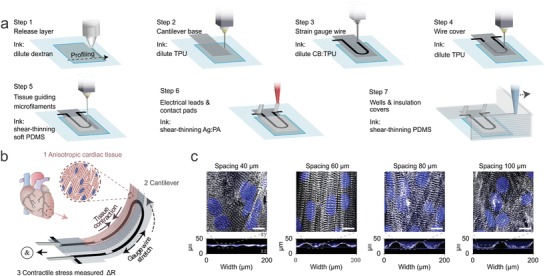
A 3D printed cardiac micro‐physiological system integrated with 3D printed sensors. a) The automated printing of multiple materials in 7 steps to generate a platform that integrated both cell constructs and sensors. b) The contraction of the anisotropic tissue leads to deflection of the cantilever, where an electrical signal was detected. c) The optimization of the microfabricated grooves for aligning cardiac cells. Reproduced with permission.^150^ Copyright 2018, Springer Nature.

With cells included into the printing inks, 3D bioprinting emerged and adopted the advantages of the geometrical precision in conventional 3D printing. The technique of growing differentiated cells in an organized 3D manner is paving the way for bringing replacement therapies for various tissues types, such as cardiac tissue.^146^ The prospect of growing tissues with patient‐derived cells could mitigate the concern of immune rejection, expanding the pool of available tissues for organ transplantation.^147^ Computer‐aided 3D bioprinting has facilitated the construction of complex tissue structures through layer‐by‐layer deposition of cell‐laden scaffolds or free cells.^148^ Similar printing techniques, such as inkjet, laser, extrusion, acoustic, and stereolithography, were used in bioprinting. Biomaterials with favorable printing properties, such as shear‐thinning, facile gelation after printing, and appropriate yield stress, are preferred as bioinks. In general, 3D bioprinters have the ability to inject printable biomaterials, or even cells in the form of sheets or spheroids, with micrometer precision.^149^ Using a custom‐made 3D bioprinter, Lind et al. printed multiple materials sequentially (**Figure** **7**a) and created devices that i) guided the alignment of cardiac cells and ii) generated sensors that can monitor the contraction of the cardiac tissue (Figure 7b,c).^150^ The 3D prototypes with elaborate internal and external structures can be rapidly built with controllable shape, porosity, and mechanical properties.^151^ With the assistance of biomedical imaging, personalized tissues with customized size and shape were printed.^152^ However, immune rejection of the artificial tissue is still a concern for these scaffolded tissue structures. The incorporation of patient‐derived starting materials, such as autologous cells^148^ or growth factors,^153^ into the bioinks helps reduce the rejection and guides printed tissues a step closer toward realizing precision medicine.

Injectable biomaterial scaffolds are the fundamental building blocks for creating 3D replacement for regenerative therapy.^154^ Many investigations have studied the physical and biochemical effects of the scaffolds on the behavior of the cells, including stem cell differentiation, migration, or viability.^155^ In the ideal case, the implanted biomaterial scaffold alone is able to attract endogenous resources from the patient to regenerate the tissue. Besides synthetic materials, natural materials such as the ECM residue resulting after decellularization of the tissue provide a structurally authentic scaffold for tissue regeneration. These decellularized scaffolds maintain their shape, size, chemical, and biological characteristics of the original tissue, which is very challenging for synthetic scaffolds to recapitulate.^156^

Another challenge for engineering thick tissues is vascularization, which supplies nutrients and oxygen to the cells and removes waste from the tissues.^157^ Microfabrication techniques using microfluidics are capable of generating controllable microscopic structures, making microfluidics a versatile tool for tuning the engineered tissues.^158^ Hollow microfibers or hydrogels prepared with soft lithography could be lined with vascular endothelial cells on the inner wall to simulate the native vasculature.^159^ Similarly, 3D printing can be integrated with a glass filament‐mediated molding to generate in vitro vasculature.^160^ Additionally, 3D printing offers solutions to this challenge through either printing porous acellular scaffolds or forming vasculature by depositing layers of different stable materials like fugitive.^161^

Currently, the prospect of engineering entire functional organs, such as lungs or hearts, is not yet realizable. Some architecturally simple tissues, like skin, blood vessels, cartilage, or bone, are much easier for tissue engineering to replicate.^162^ With patient‐derived stem cells and somatic cells, the engineered tissues can be personalized, taking yet another step toward tissue level precision medicine. Furthermore, quality control over the risks of genetic mutations, tumorigenesis, or functional instability still needs to be realized in place for translating engineered tissues to patients.

### 2.5 Organs‐on‐Chips for Precision Drug Screening

Although genomics helps determine the optimal anticancer drug of choice for each individual and there have been exceptional reports along these lines,^163^ pairing a genetic mutation with a drug could only occur in 2–6.4% of the patients.^164^ Information about the origin of a cancer by studying its native tissues could provide more direct information related to its unique characteristics.^165^ Expanding patient‐derived cancer tissues in vitro and then directly testing their responses to available anticancer therapies provide an alternative method to optimize precision anticancer prescriptions. The key to this approach is that the “avatars” need to capture the physiological traits of patient's tissue in vitro based on their histological structures and gene expression profiles.^166^ Culturing 2D established cancer cell lines is a routine method in preclinical anticancer drug development. However, the cell lines cannot faithfully capture the genotypes of a specific patient's cancer, as the heterogeneity of the cell line differs from the that of the tumor,^167^ and the 2D cellular response to the tested drug is very different from the actual scenario in vivo.^168^ By comparison, there are many advantages to the 3D organoid‐based approaches: only a small amount of patients' tissue is needed to generate the required construct in vitro,^169^ rare tumor types specific to the patient can be recreated, cell heterogeneity of the tumor is captured, drug resistance behavior similar to the tumor is simulated, toxicity and genetic profiling analyses could be performed on the cultured tumor mass, and effective drugs can be prescreened for patients.^170^ For a typical mice‐based tumor xenograft method, the organoid based approach is more suitable for high‐throughput drug screening due to its low cost, rapid in vitro tumor growth, and avoiding ethical issues regarding animal use.^171^

**Figure 8:**
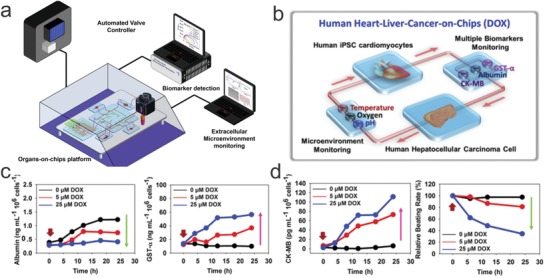
Automated sensing of cardiac toxicity of an anticancer drug using multi organoids on a chip. a) An automated system that integrates computer‐controlled fluidics, organs‐on‐chips platform, and in line sensing. b) Design of a heart–liver‐cancer‐on‐chip system to monitor the on‐target and off‐target toxicity of DOX. c) In‐line monitoring of the anticancer efficacy of DOX to liver cancer organoid. d) In‐line monitoring of the cardiac toxicity of DOX using biosensors. Reproduced with permission.^177^ Copyright 2017, National Academy of Sciences.

Biopsies from liver cancer,^172^ gastrointestinal cancer,^173^ head and neck squamous cell carcinoma,^174^ ductal pancreatic cancer,^175^ and prostate cancer^176^ have been used to create organoids in vitro. Genetic profiling has shown the recapitulation of the source cancer tissue, a viable strategy for the screening of anticancer drug candidates. It has been demonstrated that conserving the heterogeneity of the tumor microenvironment is a key factor to accurately predicting the efficacy of a drug. From an engineering perspective, the “Organs‐on‐a‐Chip” approach, integrating organoid culture and organoid behavior monitoring into one system, can enable high throughput drug testing platforms for precision medicine to be developed. For example, Zhang et al. demonstrated an organs‐on‐chips system that integrated computerized microfluidics, sensors, and tissue models for real‐time monitoring drug‐induced responses (**Figure** **8**a,b).^177^ In a heart–liver‐cancer model, anticancer efficacy of Doxorubicin (DOX) (Figure 8c) as well as cardiac toxicity caused liver‐mediated metabolism of DOX (Figure 8d) was simulated. However, current organoid chips are labor intensive, not high throughput and lack sensing methods. Further development should be made to focus on the miniaturization of the components on an integrated chip. In light of the increasingly high cost of developing new drugs, the use of the “Organs‐on‐a‐Chip” approach for prescreening the efficacy and toxicity of new drugs could be a time‐ and money‐saving strategy. In addition, the “Organs‐on‐a‐chip” approach can be applied to be expanded to study various types of cells, including both diseased and normal cells, for investigating metabolic and signaling pathways.^178^

A biopsy is the standard method for acquiring tissues from patients. In the case of cancer metastasis and circulating cancers, capturing the metastatic cells from blood and concentrating them for analysis can acquire disease‐specific information about these tissues. Circulating tumor cells (CTCs) may be less invasive than the primary tumor to obtain, and they could help in staging cancer and improving prognoses.^179^ Finding methods for specifically capturing the targeted cancer cells from the blood circulation is the key challenge for acquiring CTCs. Through utilizing properties specific to CTCs, such as overexpressed biomarkers or differences in density, size, or charge, many methods have been developed for CTC separations.^180^ Often, a combination of immunoaffinity and magnetic‐ or fluorescence‐based separations is widely used for isolating CTCs; a representative example of this is the FDA‐approved CellSearch system that captures epithelium sourced CTCs. Microfluidic devices with size exclusion or antibody‐modified channels have been demonstrated as a convenient way of isolating CTCs as well. The capturing efficacy of these channels could be fine‐tuned by optimizing the flow rate, turbulence, and variety/density of antibodies. Furthermore, by using transparent devices, imaging and capturing could be done simultaneously.

Captured CTCs can be used for directing genetic profiling or growing organoids before analysis. Besides CTCs, however, the capture of other circulating cells, such as trophoblasts, has been demonstrated to provide prenatal diagnostics.^181^ Capturing other biocomponents from the blood, such as exosomes^182^ or circulating DNA,^183^ also provides additional personalized molecular information about a patient. However, not all target cells are circulating, for tissues that are not readily accessible by either liquid or solid biopsies, differentiating cell lines in vitro using iPSC‐based technology offers an alternative method to access them. For example, iPSC‐derived cardiomyocytes have been used for testing drug toxicity.^184^

## 3 Future Directions and Challenges

In a hypothetical scenario, when a patient with breast cancer is hospitalized, the last thing she would want is to try different anticancer therapeutics, accumulating all the toxic side effects from each treatment. To tailor therapy specifically for the patient, one can run genetic sequencing on the patient's tumor tissues to acquire her genetic information and propose an optimal therapy based on the correlations between her genotype and phenotype. Meanwhile, one can also use engineering approaches to validate what this patient needs experimentally. For example, one can use biosensing techniques to test expressed biomarkers in real time to monitor her disease's progression; one could take a biopsy of the tumor and build a tumor‐on‐chip device to rapidly test the response of the tumor to different therapies so that one does not need to rely on the accuracy of bioinformatic predictions alone; and one can engineer smart biomaterials that control the delivery and release of an anticancer drug specific to the patient's tumor microenvironment so that one dose of the drug could fight cancer for weeks or months.

With more emerging advances and breakthroughs in engineering techniques and basic biology,^185^ more controllable tools will become available to increase the accuracy and application areas of precision medicine. Current bioinformatics‐based precision medicine is mostly based on our capability to read DNA. With genome editing tools like CRISPR‐Cas9 commercially available,^186^ it is now a routine laboratory technique to write predesigned DNA sequences into cells. The prospect of treating hereditary diseases from their genetic roots sheds light on many untreatable diseases.^187^ Many exploratory studies are ongoing to integrate CRISPR into therapies to correct monogenic diseases.^188^ This technology is readily integrated with regenerative medicine by editing stem cells or with cancer immunotherapy by engineering T cells, but it is not yet ready to be directly applied to humans. With the challenges of delivery efficiency^189^ and the debate over off‐targeting,^190^ new medicines with nucleotide‐level precision are expected.

Current methods to build artificial tissues from cell culture are far from ready for clinical applications in terms of wanting to generate anatomically and functionally meaningful organs. The other “top‐down” approach that works toward directly growing artificial organs in animals to generate functional tissues is more straightforward. In one example, endogenous retroviruses in porcine were systemically knocked out with the CRISPR‐Cas9 system to obviate the concern of transmitting these viruses from porcine animals to humans.^191^ Although immune compatibility issues have not been addressed in these studies yet, the approach of generating humanized pig organs are promising for producing structurally complex organs for xenotransplantation.

Effects from the environment have long been recognized as important regulators of human health; recent progress has revealed insight about our interactions with the environment into greater detail. An important venue that humans interact with the environment is through microorganisms. Traditional human‐associated microbiology studies focused on pathogenic microbes;^192^ moreover, recent studies have revealed that nonpathogenic microbes are also known to affect an individual's health, such as his or her immune system or metabolism.^193^ The composition of microorganism inside the human body or the microbiome differs significantly among people, leading to variations in the strength of an individual's immune system as well as reactions to a given drug. Infants experience their first microbiome though mother–infant transmission in the birth canal; this may be the first training of the newborn immune system to adapt to a world full of microbes. It has been found that infants missing this training by cesarean‐mediated births show weaker immune systems. Furthermore, the activity of the local microorganisms changes the metabolic pathways of a given drug, changing its pharmacokinetics. It has been recently validated that intestinal microbiome composition can severely affect the therapeutic efficacy of anticancer drugs.^194^

Health monitoring using wearable, implantable, and point of care sensors represents a future trend for achieving real‐time healthcare management. We expect that these sensors will be incorporated into every aspect of our life by bridging the “internet of things” with personalized healthcare. These sensors are already in the smartwatches we wear, and soon, they will be in other wearable gears. Perhaps we will see smart cars that not only self‐navigate but also keep an eye on the health status of the driver. They might automatically pull over to a rest area when the driver is fatigued. Furthermore, we expect smart homes with healthcare sensors integrated within furnitures or appliances. The refrigerator might give a suggestion of a grocery list to order a balanced diet for an individual, or the air conditioner might detect pathogens within a person's breath and send out warnings.

Despite the promising future of precision medicine, there are still many challenges that need to be addressed before precision medicine can fully be realized and benefit everyone.

1. In terms of customized precision medicine, there is a trade‐off between convenience and cost. From a practical perspective, the higher the degree of precision, the more complex the healthcare service will be. Similar to comparing a tailored suit to off‐the‐shelf pajamas, the more bells and whistles we incorporate into designing the device, the better the device will be, but the time and cost of fabricating it will increase, restricting everyone from having the same access to that level of care. So, it is desirable to have a panel of healthcare services that have different levels of precision to fit the need for precision medicine.
2. Engineered precision medicine will need to have an extremely high versatility to meet the needs of individual patients, increasing the complexity of gaining regulatory approvals. Using the classic DOX‐loaded liposome (Doxil) as an example,^195^ we may customize many personalized Doxil derivatives that target different receptors and trigger drug release in response to the various physiological environmental factors in different patients. Besides the manufacturing cost for the diverse product lines, getting each of the personalized medicines approved would be a daunting task. Furthermore, prescreening patients to fit a particular therapy might contradict traditional clinical trials that require randomized samples.^196^ To address this challenge, engineers could either simplify the design or incorporate mainly FDA‐approved components into their formulation. In the meantime, regulatory innovations are needed to facilitate the approval of new drugs.
3. Our current knowledge about human physiology only applies precision medicine to a tiny portion of healthcare related issues. For example, our understanding of the correlation between biomarkers that can be detected from body fluids and related diseases is still limited.^197^ More fundamental research about the mechanism of many diseases is needed, which requires us to invest more in fundamental researches.
4. Compared to traditional medicine, more well‐trained practitioners with training in genomics and engineering will be needed. In order to use devices based on engineered biomaterials, the clinicians will need to have a general understanding of various materials. All these add‐on requirements make it possible for licensing and board certification exams to include genomic‐ or engineering‐related topics. To facilitate incorporation of the new requirements, it is better to adapt current genomics and engineering trainings designed for professional geneticists and engineers to fit the need of clinicians. By focusing on essential skills or knowledge needed to apply genetic discoveries or engineered devices into the clinic, it could reduce the load on medical students but also enables them to harness the advantage of precision medicine.
5. Although most people may agree to share real‐time data from therapeutic devices, such as blood glucose monitoring, some may have concerns about sharing their static data, like their genetic information. They may also have concerns about being discriminated against for job applications based on their genetic‐information, if the information is made available to the decision maker, i.e., the employer. To address the privacy concerns from the general public but still make the sensitive informative available to the research community, it is necessary to build a data‐share system with different levels of privacy settings. Strong network security needs to be in place to prevent unauthorized access to the information.
6. Precision medicine is based on our capability to manage “Big Data,”^198^ including genomics, sensing, imaging, and other available health records. In the long run, we should be able to keep people's health records from birth to death as well as from a molecular to societal level as well. For the data to be “big” enough, healthcare data from patients with different ethnic backgrounds are needed. For example, African descendants are currently underrepresented in terms of data collection. Increasing the diversity of collected data from people of different races could spread the benefits of precision medicine to more people.^199^ The use of artificial intelligence techniques in assisting “Big Data” management for personalized medical care is promising, as it potentially holds a high level of precision and accuracy in disease diagnostics, history, treatment, and prognosis.^200^

Overall, precision medicine is an ambitious approach that needs collective efforts from physicians, patients, insurance companies, information technology developers, bioengineers, and others. It requires knowledge and technologies from various fields, such as medicine, genetics, chemical engineering, materials engineering, bioengineering, and pharmaceuticals, and people all around the world need to contribute to make it a reality. In order to transform our current healthcare infrastructure into the era of precision medicine, we need to remove many of the barriers between people from different areas. New drugs, devices, etc., are needed to take this endeavour to the next level.

## Conflict of Interest

The authors declare no conflict of interest.
